# Early treatment response as predictor of long-term outcome in a clinical cohort of children with ADHD

**DOI:** 10.1007/s00787-023-02158-z

**Published:** 2023-02-16

**Authors:** Tine Bodil Houmann, Kristine Kaalund-Brok, Lars Clemmensen, Morten Aagaard Petersen, Kerstin Jessica Plessen, Niels Bilenberg, Frank Verhulst, Pia Jeppesen, INDICES, Henrik Berg Rasmussen, Ditte Bjerre, Majbritt Busk Madsen, Laura Ferrero, Kristian Linnet, Ragnar Thomsen, Gesche Jürgens, Claus Stage, Hreinn Stefansson, Thomas Hankemeier, Rima Kaddurah-Daouk, Søren Brunak, Olivier Taboureau, Grace Shema Nzabonimpa, Tine Houmann, Pia Jeppesen, Kristine Kaalund-Brok, Peter Riis Hansen, Karl Emil Kristensen, Anne Katrine Pagsberg, Kerstin Plessen, Poul-Erik Hansen, Wei Zhang, Thomas Werge

**Affiliations:** 1 Child and Adolescent Mental Health Center, Mental Health Services-Capital Region of Denmark, Copenhagen, Denmark; 2 Copenhagen Research Centre on Mental Health (CORE), Copenhagen University Hospital, 2900 Hellerup, Denmark; 3 Palliative Care Research Unit, Department of Geriatrics and Palliative Medicine GP, Bispebjerg & Frederiksberg Hospital, University of Copenhagen, Copenhagen, Denmark; 4 Service Universitaire de Psychiatrie de L’Enfant Et de L’Adolescent, Centre Hospitalier Universitaire Vaudois, University of Lausanne, Lausanne, Switzerland; 5 Department of Child and Adolescent Mental Health Odense, Research Unit (University Function), Mental Health Services in the Region of Southern Denmark, Odense, Denmark; 6 Department of Clinical Medicine, Faculty of Health and Medical Sciences, University of Copenhagen, Copenhagen, Denmark; 7 Department of Child and Adolescent Psychiatry, Copenhagen University Hospital-Psychiatry Region Zealand, Smedegade 16, 4000 Roskilde, Denmark

**Keywords:** ADHD, Treatment response, Predictor, Long-term outcome

## Abstract

This study investigates early onset of treatment response as predictor of symptomatic and functional outcome 3 years after initiation of methylphenidate (MPH) administration in a naturalistic, clinical cohort of children and adolescents with ADHD. Children were followed across an initial 12-week MPH treatment trial and after 3 years, with ratings of symptoms and impairment. Associations between a clinically significant MPH treatment response in week 3 (defined as ≥ 20% reduction in clinician-rated symptoms) and in week 12 (defined as ≥ 40% reduction), and 3-year outcome were tested in multivariate linear regression models, adjusting for sex, age, comorbidity, IQ, maternal education, parental psychiatric disorder, and baseline symptoms and function. We did not have information on treatment adherence or the nature of treatments beyond 12 weeks. 148 children, mean age 12.4 years (range 10–16 years), 77% males, participated in the follow-up. We found a significant decrease in symptom score from baseline [*M* = 41.9 (SD = 13.2)] to 3-year follow-up [*M* = 27.5 (SD = 12.7), *p* < 0.001, and in impairment score from baseline (*M* = 41.6 (SD = 19.4)] to 3-year follow-up [*M* = 35.6 (SD = 20.2), *p* = 0.005]. Treatment responses in week 3 and week 12 were significant predictors of the long-term outcome of symptoms, but not of impairment at 3-year follow-up, when adjusting for other well-known predictors. Early treatment response predicts long-term outcome over and above other well-known predictors. Clinicians should follow-up patients carefully, during the first months of treatment, and detect non-responders, since there might be a window of opportunity to alter the outcome, by changing the treatment strategy.

**Clinical trial registration:** ClinicalTrials.gov, registration number NCT04366609, April 28, 2020 retrospectively registered.

**Supplementary Information:**

The online version contains supplementary material available at 10.1007/s00787-023-02158-z.

## Introduction

Methylphenidate (MPH) is the first-line medication [11] in treatment of ADHD in children and adolescents. The evidence for the short-term effect is well established, based on randomized controlled trials (RCT’s). In contrast, only weak evidence exists concerning its long-term effect, due to ethical and methodological challenges in conducting long-term RCTs. Register and cohort studies using a within-individual design combine information of ADHD medication use, incidents of risk- and suicidal behavior, and academic performance during medicated and nonmedicated periods, within the same individual. These studies suggest a decreased risk during medicated periods regarding accidents, suicidal behavior, substance abuse, and delinquency [9, 12, 22, 27, 29], and a positive effect on student grade point average [17]. In spite of this, the adult follow-up in the Multimodal Treatment of Attention Deficit Hyperactivity (MTA) Study [31], and other follow-up studies in late adolescence [30, 33] have demonstrated that the long-term outcome in childhood ADHD is independent of treatment with ADHD medication during the follow-up period. Moreover, the long-term compliance to ADHD medication is weak [6, 31], with perceived effectiveness of ADHD medication and systematic titration of medication being some of the factors associated with a better compliance to medication [16]. A few short-term studies have investigated predictors of the immediate effect of treatment with MPH and reported that higher baseline severity of inattention and higher response time variability on continuous performance tests were associated with a poorer response to medication with MPH, measured as mean composite score on the Continuous Performance Test [26] and as total score on the Korean ADHD rating scale [21].

Early treatment response as predictor was studied in a double-blind placebo controlled trial of MPH treatment in children with ADHD [7] which described that a positive behavioral change after the first single dose of MPH was the strongest predictor of improvement in multiple settings after 4 weeks of treatment.

Since adherence to ADHD medication is weak, it is difficult to disentangle predictors of long-term outcome of the disorder from predictors of long-term treatment effects in ADHD. Increased severity of ADHD symptoms in childhood, psychiatric comorbidity, and family adversities have consistently been identified as predictors of a poorer outcome of childhood ADHD. Higher levels of parent-reported impairment in daily and social functioning, female sex, and lower IQ of the child have also been associated with poorer long-term outcomes [8, 10, 20, 28, 33].

This study aimed to extend the evidence base for long-term symptomatic and functional outcome of MPH treatment, and the predictors of outcomes focusing on the early treatment response as predictor in 7–12-year-old children with a recent diagnosis of ADHD and first initiation of treatment with MPH. The association between the markers of early response and the long-term outcomes were adjusted for potential confounders. Our overarching aim was to investigate the potential for clinical use of instrument-based assessments and evaluation of the early and individual course of response to MPH treatment, as a strategy for improved short- and long-term outcomes in clinical care. More specifically, we tested if an early treatment response versus non-response to MPH predicted the long-term outcome measured as ADHD symptoms and level of daily functioning.

We tested the following hypotheses:

- The early treatment response, measured as reduction in symptoms at 3 weeks and 12 weeks, would be associated with the symptomatic outcome after 3 years.
- The early treatment response, measured as reduction in symptoms at 3 weeks and 12 weeks, would be associated with the functional outcome after 3 years.
- The associations would be robust after adjustment for known predictors (gender, age, comorbidity, IQ, maternal educational level, and parental psychiatric disorders), and for baseline level of symptoms and baseline level of impairment.

## Methods

### Participants

The study is a follow-up of participants in the INDICES study, a prospective longitudinal 12-week ecologically valid observational study of first treatment with methylphenidate in a representative clinical sample of drug naïve children [19]. The study was conducted as part of the routine care in the clinic, and the individually monitored treatment with MPH was part of the study by the INDICES consortium aiming at personalizing the treatment of drugs metabolized by CES1 [5]. Patients were recruited from the Child and Adolescent Mental Health Centre, Mental Health Services, Capital Region of Denmark from August 2011 to December 2014. Participants at baseline included a total of 207 children (75.4% boys), aged 7–12 years (mean age 9.58 years), with a recent ICD-10 diagnosis of hyperkinetic disorder (F90.0–90.9), or attention-deficit disorder without hyperactivity (F98.8), IQ ≥ 70, and clinical indication for treatment with IR-MPH. The exclusion criteria were former treatment with any ADHD medication (MPH, dexamfetamine, lisdexamfetamine, or atomoxetine), contraindication for treatment with MPH, Danish language at a level that did not allow a valid exploration, or lack of informed consent.

For the 3-year follow-up, the parents and/or legal guardians of all 207 participant at baseline were contacted by letter, or later by telephone if they did not respond. They were informed of the study and invited to participate in the 3-year follow-up, by answering two questionnaires included in the contact letter. Only participants with data at baseline, week 3, week 12, and after 3 years are included in the analyses.

**Fig. 1:**
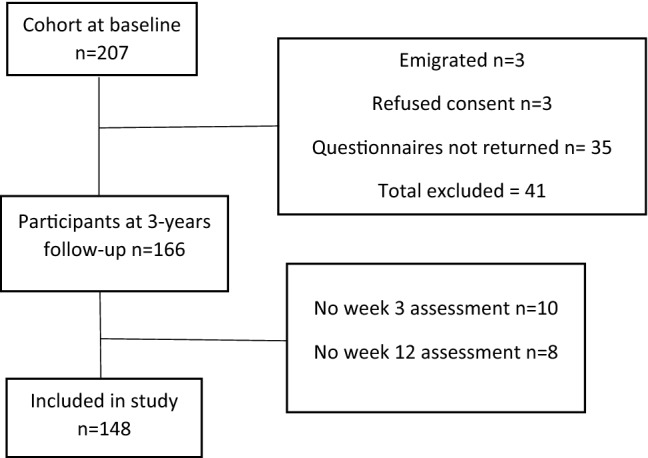
Flowchart of inclusion of participants from baseline to 3-year follow-up

Figure 1 shows the flowchart of participants from baseline to the 3-year follow-up.

### The 12-week MPH treatment trial and assessment procedures

Participants received an initial Immediate Release (IR) MPH dose (2.5/5 mg) based on bodyweight (< / > 30 kg), two or three times a day according to participants individual symptoms and needs. The dosing was individually titrated based on weekly assessments of effect and adverse reactions (AR), until AR prohibited further MPH dose increase, or cut-off for the normal range or borderline range on the ADHD-Rating Scale (ADHD-RS) was achieved. ADHD-RS has been validated in a Danish general population-based sample (*n* = 865 children) [32]. We used the standardized scores (t-scores) stratified for each sex and age group (7–9 and 10–12 years) to delineate the cut-off for the normal range (≤ 60 *t*-scores) or borderline range (60–70 *t*-scores) on the clinician-rated ADHD-RS-Clinician (ADHD-RS-C) total score (inattention plus hyperactivity–impulsivity scores) and scores of inattention and hyperactivity–impulsivity respectively, in the present study [13, 32, 37]. The study had a pre-defined MPH maximum dose of 2.1 mg/kg/day. The mean IR-MPH end-dose after 12 weeks was 1.0 (0.3), (range 0–1.79) mg/kg day.

During the trial, participants were assessed weekly, with ratings of ADHD symptoms (ADHD-RS-C) and AR’s on the Barkley’s Stimulant Side Effect Rating Scale (BSSERS) [4] conducted by the clinical investigator and the child’s regular clinician, based on telephone interviews with the parents, and clinical assessments at baseline, week 4, 8, and 12. These included physical examination, observation of the child, interview with the parents, and consensus ratings on the ADHD-RS-C. ADHD and disruptive behavior symptoms were rated by parents at baseline, and at 12-week follow-up, on the ADHD-Rating Scale Barkley version (ADHD-RS) [3]. Impairment in daily life- and social functioning was rated by parents on the Weiss Functional Impairment Rating Scale (WFIRS-P) [34] at baseline and at 12-week follow-up.

### The 3-year follow-up

Three years after completing the 12-week MPH treatment trial, long-term outcome was evaluated by the parent-reported severity of ADHD and disruptive behavior symptoms, and impairment in daily life- and social functioning, using the ADHD-RS and the WFIRS-P questionnaires. We did not have information concerning use of ADHD medication, non-medical treatments, and adherence to treatment from week 12 to 3-year follow-up.

### Outcome variables

The ADHD-RS-C consists of 18 clinician-rated questions evaluated on a four-point Likert scale from 0 (none = never or rarely) to 3 (severe = very often). It measures ADHD symptoms including a total score and two subscales: inattention (9 items) and hyperactivity–impulsivity (9 items). Thus, the total score ranges from 0 to 54. Higher scores indicate worse outcomes.

The ADHD-RS Barkley version is like the ADHD-RS-C but includes a behavior subscale. It consists of 26 parent-rated questions. In addition to the inattention- and hyperactivity–impulsivity subscales, it includes a disruptive behavior subscale (8 items). Thus, the total score ranges from 0 to 78. Higher scores indicate worse outcomes.

The WFIRS-P measures children's daily life- and social functioning. It consists of 50 parent-rated questions evaluated on a four-point Likert scale from 0 (never or not at all) to 3 (very often or very much). WFIRS-P covers six different domains: family (10 items); learning and school (10 items); activities of daily living (10 items); self-concept (3 items); social activities (7 items); and risky activities (10 items). Thus, the total score ranges from 0 to 150. Higher scores indicate worse outcomes. WFIRS-P has been validated in multiple cultures [35], including a recent Norwegian validation study [15].

### Predictor variables

Treatment response after 3 weeks was defined as ≥ 20% reduction in clinician-rated ADHD-RS-C total score, between baseline and week 3. Treatment response after 12 weeks was defined as ≥ 40% reduction in clinician-rated ADHD-RS-C total score between baseline and week 12*.*

In addition, we included sex, age group divided in young (7–9 years) and old (10–12 years), comorbidity (none versus ≥ 1 comorbid diagnosis), IQ (full scale IQ 70–85/ > 85), and parental psychiatric disorder (none/ any maternal or paternal disorder). As a proxy measure of socio-economic status, we used maternal education.

Information of any parental lifetime psychiatric diagnoses was obtained from the Danish Psychiatric Central Register. Information of mother’s educational level was obtained at baseline by interview with the parent and categorized in three groups ranging from primary school to a longer higher education at university level, based on years of schooling and education (primary/lower secondary education, upper secondary education, higher education).

### Ethics

The study was registered in ClinicalTrials.gov (NCT04366609). The study was approved by the Danish Data Protection Agency (P-2019-851). The Local Committee on Health Research Ethics was consulted (J.nr. H-B-2009-026) in accordance with national guidelines and the Declaration of Helsinki, and the study was evaluated not to be within their jurisdiction due to study design as an observational study. Participation was voluntary and data were kept confidential. The participants could withdraw their consent at any time without having to give reasons and with no consequences for their further treatment options.

### Statistical analyses

Attrition analyses were performed to explore potential differences in baseline characteristics, ADHD-RS-C-, ADHD-RS- and WFIRS-P scores in the 12-week treatment trial between participants in the 3-year follow-up and those who were lost to follow-up. We used Chi-square tests for categorical variables, Mann–Whitney test for ordinal variables, and independent *t *tests for continuous variables.

Missing data on any items were not allowed on ADHD-RS-C and ADHD-RS. Ten percent missing data on items on WFIRS-P subscales were allowed, and missing data on items were set as 0 (never or not at all).

Paired-samples *t *tests were performed to explore the development of ADHD-RS and WFIRS-P scores from baseline, after 12 weeks of MPH treatment to 3-year follow-up.

Two-sample *t *tests were applied to test associations between a positive MPH treatment response in week 3 (≥ 20% reduction in symptom score) and in week 12 (≥ 40% reduction in symptom score), respectively, and outcome on the parent-rated ADHD-RS total score and WFIRS-P total score after 3 years.

Multiple linear regression analyses were conducted to test the effect of adjusting the crude associations obtained with two-sample *t *tests, for the selected predictor variables. Quantile plots [36] of the outcome variables in these multiple linear regression analyses (ADHD-RS total- and three subscales scores and WFIRS-P total score) were performed to assess the assumption of normally distributed outcomes. They showed approximately normal distribution for all variables. To explore the effect of including either treatment response at week 3, or treatment response at week 12, we tested two models of predictors. Model 1 includes sex, age group, comorbidity, IQ (IQ 70–85 vs > 85), maternal education, parental psychiatric disorder, baseline ADHD-RS total score, baseline WFIRS-P total score, and responder status in week 3. Model 2 includes all the same co-variates and responder status at week 12. The reason for including two models of predictors was to explore whether treatment response could be evaluated as early as after 3 weeks. The primary outcomes were ADHD-RS total score and WFIRS-P total score. In addition, we measured outcome on the three ADHD-RS subscales. Adjusted *R*^2^ was used as a measure of the proportion of the variance in the outcome variables, explained by potential predictors in the two models.

All analyses were conducted using the IBM SPSS Statistics 25 program. All tests were two sided, and significance was set at *p* < 0.05.

## Results

### Study population

Table 1 shows the baseline characteristics of the participants in the 3-year follow-up and those who dropped out. Of the original sample (*n* = 207) 148 had ADHD-RS-C ratings at baseline, week-3, and week-12, thus constituting the study population and a retention rate of 71%. WFIRS-P rating was available for 139 participants at baseline, and 132 at 3-year follow-up. Independent *t* tests, Mann–Whitney test, and Chi-square tests showed no significant differences in individual characteristics, comorbidities, parental psychiatric disorders, maternal education, ADHD subtypes, and scores on the ADHD-RS-C, ADHD-RS, and WFIRS-P between the 3-year follow-up sample and dropouts.

**Table 1:** Baseline characteristics of participants in 3-year follow-up versus dropouts

	Participants (*N* = 148)	Dropouts (*N* = 59)
Sex, boys, *n* (%)	114 (77.0)	41 (71.9)
Age at study entry in years, mean (SD)	8.94 (1.47)	9.28 (1.48)
Age group 7–9 years at study entry *n* (%)	100 (67.6)	33 (55.9)
ADHD diagnoses (ICD-10)		
Hyperkinetic disorder (F90.0, F90.8, F90.9) *n* (%)	124 (83.8)	48 (81.4)
Hyperkinetic conduct disorder (F90.1) *n* (%)	6 (4.1)	6 (10.2)
Attention-deficit disorder without hyperactivity (F98.8C) *n* (%)	18 (12.2)	5 (8.5)
Comorbidity		
≥ 1 comorbid diagnosis, *n* (%)	86 (58.1)	37 (62.7)
Mean (SD), range	1.03 (0.9), 0–4	1.03 (0.9), 0–4
Externalizing disorders^a^ *n* (%)	12 (8.1)	2 (3.4)
Emotional disorders^b^ *n* (%)	17 (11.5)	10 (16.9)
Autism spectrum disorders *n* (%)	22 (14.9)	4 (6.8)
IQ		
WISC-IQ > 85 (normal), *n* (%)	107 (72.3)	44 (74.6)
Maternal educational level		
Primary and lower secondary education^c^, *n* (%)	17 (11.6)	7 (13.0)
Upper secondary education^d^, *n* (%)	51(34.9)	22 (40.7)
Higher education^e^, *n* (%)	78 (53.4)	25 (46.3)
Parental psychiatric disorder		
One or more diagnoses, *n* (%)	38 (25.7)	15 (31.3)
ADHD-RS-C^f^ total score		
Week 0, mean (SD)	37.5 (8.1)	39.0 (6.7)
Week 3, mean (SD)	31.3 (9.0)	32.8 (7.7)
Week 12, mean (SD)	17.6 (6.9)	17.2 (6.2)
ADHD-RS^g^ total score		
Week 0, mean (SD)	41.9 (13.1)	39.9 (15.4)
Week 12, mean (SD)	23.7 (10.9)	24.3 (15.0)
WFIRS-P^g^ total score		
Week 0, mean (SD)	41.6 (19.5)	40.6 (20.2)
Week 12, mean (SD)	29.4 (14.5)	29.4 (14.5)

^a^Conduct disorders, mixed disorders of conduct and emotions

^b^Mood, anxiety, adjustment disorders, OCD

^c^Up to 10 years

^d^11–14 years

^e^15–19 years

^f^Clinician rated

^g^Parent rated

### The course of ADHD symptoms and impairment in daily life- and social functioning over time

ADHD symptoms, measured by the ADHD-RS total score, decreased significantly from baseline [*M* = 41.9 (SD = 13.2)] to 12-week follow-up [*M* = 23.7(SD = 10.9), *p* < 0.001], followed by a slight but significant increase in score at 3-year follow-up [*M* = 27.5(SD = 12.7), *p* < 0.001].

Impairment in daily life and social functioning, measured by WFIRS-P total score, decreased significantly from baseline [*M* = 41.6 (SD = 19.4)] to 12-week follow-up [*M* = 29.4 (SD = 14.5), *p* < 0.001], indicating better functioning, followed by a slight but significant increase in score at 3-year follow-up [*M* = 35.6 (SD = 20.2), *p* = 0.005].

### Early treatment response as predictor of long-term outcome (unadjusted analyses)

Analyses of the association between treatment response at 3 weeks (≥ 20% reduction in ADHD-RS-C total score) and 12 weeks (≥ 40% reduction in ADHD-RS-C total score), and symptomatic and functional outcome at 3-year follow-up showed the following results: week-3 responders (*N* = 56) had a significantly lower ADHD-RS-total score at 3-year follow-up [*M* = 24.1 (SD = 11.0)], compared with week-3 non-responders [*M* = 29.6 (SD = 13.3), *t*(146) = 2.632, *p* = 0.009]. Additionally, responders at week 12 (*N* = 121) had a significantly lower ADHD-RS-total score [*M* = 26.0 (SD = 12.3)] compared with week-12 non-responders [*M* = 34.2 (SD = 12.5) *t*(146) = 3.097, *p* = 0.002] at 3-year follow-up. There was no significant difference between week-3 responders [*M* = 31.9 (SD = 19.1)] and non-responders [*M* = 37.9 (SD = 20.6), *t*(143) = 1.736, *p* = 0.085) on daily and social functioning measured with the WFIRS-P total score at 3-year follow-up. Week-12 responders, however, had a significantly lower WFIRS-P total score [*M* = 33.9 (SD = 20.1) compared to week 12-non-responders (*M* = 43.3 (SD = 18.7), *t*(143) = 2.185, *p* = 0.031] at 3-year follow-up, indicating better functioning.

### Adjusting for known predictors

Table 2 shows the results of the multivariate linear regression analyses of correlates of the ADHD-RS-total score outcome at the 3-year follow-up, adjusting the crude associations with the selected predictors in the two models, investigating the effects of the early treatment response after 3 or 12 weeks, respectively. Both remained significant predictors for better outcome after the adjustment. Female sex and young age (both models), higher baseline WFIRS-P score in model 1, and ≥ 1 comorbid diagnosis in model 2 significantly predicted worse outcome at 3 years (model 1 adj. *R*^2^ = 0.26, model 2 adj. *R*^2^ = 0.27).

**Table 2:** Predictors for outcome at 3-year follow-up based on multiple linear regression analyses

Variable	ADHD-RS total score at 3 years follow-up									
	Model 1^a^ Early treatment responders/responders at week 3					Model 2^b^ Late treatment responders/responders at week 12				
	*B*	SE B	β	*p*	*B* 95%CI	*B*	SE B	β	*p*	*B* 95% CI
Constant	10.009	6.218		0.107	− 2.21–22.41	13.980	6.609		0.036	0.90–27.06
Sex (female)	5.209	2.275	0.178	0.024*	0.70–9.71	5.172	2.265	0.177	0.024*	0.69–9.66
Age (10–12 yrs)	− 6.238	2.127	−0.226	0.004*	− 10.45 to − 2.03	− 6.545	2.115	− 0.237	0.002*	− 10.73– − 2.36
Comorbidity^c^	3.990	2.119	0.159	0.062	− 0.21–8.19	4.548	2.057	0.181	0.029*	0.48–8.62
WISC-IQ (IQ > 85)	− 1.657	2.280	− 0.058	0.469	− 6.17–2.86	− 1.720	2.269	− 0.060	0.450	−6.21–2.77
Maternal education^d^	−0.665	1.496	−0.037	0.657	− 3.63–2.30	−0.606	1.490	−0.033	0.685	−3.56–2.34
Parental psychiatric disorder^e^	− 0.169	2.271	− 0.006	0.941	− 4.66–4.33	−0.516	2.259	−0.018	0.820	−4.99–3.96
Baseline ADHD-RS total score^f^	0.193	0.102	0.206	0.060	− 0.01–0.39	0.196	0.101	0.209	0.055	− 0.004–0.40
Baseline WFIRS-P total score^f^	0.148	0.070	0.230	0.037*	0.01–0.29	0.125	0.069	0.196	0.072	−0.01–0.26
Response week 3 (≥ 20% symptom reduction), *n* = 56	− 4.029	2.003	−0.156	0.046*	− 7.90 to − 0.07					
Response week 12 (≥ 40% symptom reduction), *n* = 121						− 5.802	2.522	−0.176	0.023*	− 10.80 to − 0.81

^a^Model 1, *R*^2^ = 0.31, adj. *R*^2^ = 0.26

^b^Model 2, *R*^2^ = 0.32, adj. *R*^2^ = 0.27

^c^ ≥ 1 comorbid diagnosis

^d^Higher education

^e^Any parental psychiatric diagnosis

^f^Higher score, worse outcome

*p < 0.05

Regarding the outcome measured on the ADHD-RS subscales (Online Resource Table 2b–2d), we found no significant associations of the treatment response in week 3 or week 12 with scores of inattention at the 3-year follow-up. Young age in both models, along with higher baseline WFIRS-P score in model 1, significantly predicted worse outcome of inattention at 3 years (model 1 adj. *R*^2^ = 0.08, model 2 adj. *R*^2^ = 0.09).

Regarding the outcome measured on the hyperactivity/ impulsivity subscale, the treatment response in week 3 and week 12 remained significant predictors for better outcome at 3 years. Female sex, young age, ≥ 1 comorbid diagnosis, and higher baseline ADHD-RS total score significantly predicted worse outcome of hyperactivity/impulsivity (model 1 adj. *R*^2^ = 0.23, model 2 adj. *R*^2^ = 0.22).

For the outcome measured on the disruptive behavior subscale, the treatment response in week 12 remained a significant predictor for better outcome at 3 years, whereas there was no significant effect of the treatment response in week 3. Higher baseline ADHD-RS total score and higher baseline WFIRS-P score significantly predicted worse outcome in both models, (model 1 adj. *R*^2^ = 0.25, model 2 adj. *R*^2^ = 0.26).

Regarding the functional outcome measured as the WFIRS-P total score at 3 years (Online Resource Table 2e), neither treatment response in week 3 nor week 12 reached significance. Higher baseline WFIRS-P total score was the only significant predictor (worse outcome) (model 1 adj. *R*^2^ = 0.17, model 2 adj. *R*^2^ = 0.16).

## Discussion

In this 3-year follow-up study of a naturalistic, clinical cohort of children with ADHD, we studied the early response versus non-response to medical treatment with MPH as putative predictors for a better symptomatic and functional outcome 3 years after initiation of treatment, adjusting for other known predictors of the long-term outcome of treatment for ADHD. In summary, positive response to MPH treatment 3 and 12 weeks after initiation, measured respectively as a 20% or 40% reduction in symptoms, significantly predicted less hyperactivity/impulsivity and oppositional defiant symptoms at 3-year follow-up, over and above baseline symptoms and impairment, comorbidity, and other well-known predictors.

There may be several reasons why early treatment response can predict severity of disorder 3 years later. We did not have information of adherence to ADHD medication beyond 12 weeks in our study, but expectedly effective control of ADHD symptoms will result in less negative feedback from the environment, a better self-esteem, less peer rejection, and a better performance in school [2, 17, 23, 25]. Additionally, effective symptom control in the child will have a positive impact on family-life and parent child relationship [14, 18]. The experience of treatment failure may lead to negative expectations in the young person and the family toward medical treatment and the health-care system, resulting in non-compliance with medication and abandoned treatment in general. Contrary, an early positive treatment response may improve the working alliance between the child with ADHD, the family, and the clinician. Expectedly, this will enhance adherence to non-medical and medical treatment and support, and result in a better long-term outcome [16], which may be reflected by the results of our study. The reason could also be that there are other unknown factors associated with treatment response, that characterize aspects of ADHD or signals severity, which are not captured by the ADHD-RS scores, even after adjusting for well-known predictors.

The well-known predictors, such as sex, age, comorbidity, and severity of baseline symptoms and function, were treated as co-variates in the multivariate linear regression analyses, and most factors remained predictive, in line with other studies [10, 20, 33], whereas neither maternal educational level nor parental psychopathology were significant predictors of the outcome of the child in this treatment study. In their 6-year follow-up study, van Lieshout et al. [33] described the same result for parental educational level but showed that parental ADHD was a significant predictor. The reason for this difference could be that they used self-reported data on parental ADHD status based on the K-SADS interview, whereas we use register data, based on hospital contacts, and thus deal with more severe and less-frequent cases of parental psychopathology, which could have decreased its predictive ability. Both parental psychopathology and lower maternal education were slightly, but insignificantly more frequent in dropouts versus participants in our study, and autism spectrum disorders were more common in the group of participants compared to dropouts, though the difference was not significant. The rate of externalizing disorders was low in both participants and dropouts. This may influence the long-term outcome in the cohort and is important to notice when comparing with other studies [20, 28].

Our models of predictive variables explained as much as 27%, 23%, and 26% of the variance in 3-year outcome measured as ADHD-RS total score, hyperactivity/impulsivity score, and disruptive behavior score, respectively. Contrary to this, the predictive variables only explained up to 9% of the variance in ADHD-RS inattention score, and 17% of the variance in WFIRS total score, indicating that development of inattention and impairment in function are predicted by other factors than those under investigation in this study.

The significant decrease in severity of ADHD symptoms and impairment during the initial 12-week treatment trial, followed by a slight increase of both symptoms and impairment from week-12 to 3-year follow-up, may be explained by the fact that the systematic assessments and individually adapted medication in the trial stopped at week 12, leading to significantly less-frequent clinical follow-up evaluations of beneficial and adverse effects of medications, and daily life- and social functioning. Still, we found an overall significant decrease in symptoms and impairment from baseline to 3-year follow-up. This is in line with other long-term follow-up studies of childhood ADHD [20, 30, 33] describing clinical improvement over the course of adolescence regardless of treatment status. Participants in the study by van Lieshout et al. [33] were older at follow-up and had a larger reduction in hyperactivity–impulsivity symptoms, but the relative change scores of ADHD symptoms are difficult to compare due to differences in age, and follow-up period.

Our results indicate that clinicians must assess and treat those who do not respond to ADHD medication during the first months of treatment with bigger efforts, since they have a worse long-term prognosis, and the poorer prognosis was only partly explained by psychiatric comorbidity and other well-known predictors. If non-responders are detected early and addressed by evaluating the treatment strategy, there might be a window of opportunity to improve the long-term outcome. In cases of non-response, the clinician must consider whether the dosing or choice of ADHD medication should be altered, and whether the psychosocial support and treatment is sufficient. In cases with persistent non-response, a diagnostic re-evaluation must be considered. Children who respond well to ADHD medication should be regularly assessed according to the clinical guidelines [1], including assessment of beneficial- and side-effects, daily function in school, home and with peers, and adjustment of medical and non-medical treatment as needed.

Our results extend the evidence from the 8-year follow-up study of the MTA cohort, suggesting that the initial response to treatment is associated with a better long-term prognosis even after adjustment for the initial clinical presentation [24].

## Strengths and limitations

The main strength of this study is the 3-year follow-up of a naturalistic clinical cohort, who represent a typical population of children with ADHD and one or more comorbid disorders, treated in the Child and Adolescent Mental Health Services in Denmark. The diagnosis of ADHD and comorbidities was based on best practice and standardized instruments including the K-SADS diagnostic interview. Associations of early treatment response and 3-year outcome were adjusted for other well-known predictors in multivariate linear regression analyses.

The study has several limitations. First, we do not have any test of interrater reliability of the diagnostic assessments. Incorrect diagnoses will have implications for the 3-year outcome. Second, the observational design without a control group makes it uncertain whether the observed significant decline in ADHD symptoms during the initial 12-week treatment trial was an effect of the medication or other factors, including the weekly contact with the clinical investigator, and whether the development of symptoms and impairment at 3-year follow-up just followed the natural course of the disorder. Third, all ratings of ADHD symptoms and function were not blinded. Clinicians and parents may have been biased by their wishes and efforts to improve the child’s symptoms and functioning. Fourth, we have no data concerning use of ADHD medication and non-medical treatments from week-12 to 3-year follow-up. Fifth, ratings of symptoms and functioning at 3-year follow-up did not consider treatment status, other psychiatric symptoms, and physical health. Sixth, we only have follow-up data from one time point during 3 years. Seventh, the huge methodological variability in studies investigating the long-term outcome in clinical cohorts of children with ADHD makes it difficult to compare the results of our study with other studies.

To get a better understanding of predictors and outcome in childhood ADHD, researchers should conduct long-term randomized controlled trials of treatments in representative clinical populations of children and adolescents with ADHD, to investigate the beneficial and adverse effects of different treatment approaches, adherence, and predictors of treatment outcome. Future naturalistic studies should investigate larger cohorts and, as it is possible in Denmark and other Scandinavian countries, include information from national registries such as the extent of psychosocial treatment, medication use, school graduation exams, and lifetime psychiatric and somatic diagnoses.

## Conclusion

This 3-year follow-up of a clinical cohort of children with ADHD, without information of adherence to ADHD medication beyond 3 months, demonstrates that early treatment response at 3 and 12 weeks predicts a better long-term outcome. An early response to medical treatment after 3 weeks is a good prognostic factor, but some of the non-responders will respond at 12 weeks, suggesting that the treatment with MPH needs to be closely monitored to optimize treatment effect, including considering a switch to other medication, if MPH treatment is suboptimal of fails despite adequate dosing during the first 12 weeks of treatment.

In line with other long-term follow-up studies, we found that severity of ADHD symptoms decreased significantly during the initial treatment period with regular and frequent clinical evaluations, but the beneficial effects decreased somewhat when the frequent follow-up by clinicians stopped. Overall, the study indicates that clinicians must assess and treat those who do not respond to ADHD medication during the first months of treatment with bigger efforts, and emphasize the importance of regular clinical monitoring and evaluation of the treatment strategy in childhood ADHD.

### Supplementary Information

Below is the link to the electronic supplementary material.

Supplementary file1 (PDF 260 KB)

## Data Availability

The pseudonymous individual participant data that underlie the results reported in this article (text, tables, figures, and appendices) can be made available to investigators for individual participant data meta-analyses that have been approved by independent review committees. The data access will be granted on a case-by-case basis by the principal investigator (Tine Bodil Houmann) and the non-author point of contact, the data manager Michella Heinrichsen after further approval by the Capital Region of Denmark, Copenhagen, Denmark. Access will be granted to the extent permissible by the General Data Protection Regulation and the Danish Data Protection Act. Making the data available may require approval from the Danish Data Protection Authority. The pseudonymous data can be made available from 6 months after the publication date of this Article, and with no end date. Proposals for use of data and requests for access should be directed to tine.houmann@regionh.dk or michella.heinrichsen@regionh.dk. To gain access, researchers will need to sign a data access agreement with the Research Unit of the Child and Adolescent Mental Health Centre—Capital Region of Denmark, Copenhagen, Denmark. *Software application:* IBM SPSS Statistics version 25.0. Armonk, NY, USA: IBM Corp. 2017.
