# Supplementary material for: Early treatment response as predictor of long-term outcome in a clinical cohort of children with ADHD
Source: Eur Child Adolesc Psychiatry. 2023 Feb 16;33(2):357–67. doi: 10.1007/s00787-023-02158-z (PMC10869385; doi:10.1007/s00787-023-02158-z)
Supplement: Supplementary file 1 — Supplementary file1 (PDF 260 KB) [file 787_2023_2158_MOESM1_ESM.pdf]

Supplementary information

European Child and Adolescent Psychiatry

Early treatment response as predictor of long-term outcome in a clinical cohort of children with ADHD

Tine Bodil Houmann ORCID 0000-0002-6231-2478, Kristine Kaalund-Brok ORCID 0000-0002-9686-1846, Lars Clemmensen ORCID 0000-0002-1750-4649, Morten Aagaard Petersen, Kerstin Jessica Plessen ORCID 0000-0001-5233-6145, Niels Bilenberg ORCID 0000-0002-5838-556X, Frank Verhulst ORCID 0000-0003-4871-2210, Pia Jeppesen ORCHID 0000-0003-2471-2929, INDICES

Corresponding author:

ORCID 0000-0002-6231-2478, tine.houmann@regionh.dk

Child and Adolescent Mental Health Center, Mental Health Services – Capital Region of Denmark, Copenhagen, Denmark

Table 2b-e Predictors for outcome at 3-year follow-up based on multiple linear regression analyses

Table 2b

	**ADHD-RS inattention score at 3-year follow-up**									
	Model 1[a] Early treatment responders/responders at week-3					Model 2[b] Late treatment responders/responders at week-12				
Variable	**B**	**SE B**	**β**	**p**	**B 95%CI**	**B**	**SE B**	**β**	**p**	**B 95% CI**
Constant	8.484	2.881		.004	2.78-14.18	9.539	3.070		.002	3.46 – 15.61
Gender (female)	1.173	1.054	.096	.268	-0.91 – 3.26	1.159	1.052	.095	.273	-0.92 – 3.24
Age (10-12 yrs)	-2.728	-986	-.237	.007*	-4.68 – -0.78	-2.800	.982	-.243	.005*	-4.75 – -0.86
Comorbidity[c]	.630	.982	.060	.522	-1.31 – 2,57	.744	.955	.071	.438	-1.15 – 2.64
WISC-IQ (IQ > 85)	-.084	1.056	-.007	.937	-2.18 – 2.01	-.102	1.054	-.009	.923	-2.19 – 1.98
Maternal education[d]	-.197	.693	-.026	.777	-1.57 – 1.18	-.177	.692	-.023	.799	-1.55 –1.19
Parental psychiatric disorder[e]	.103	1.052	.009	.922	-1.98 – 2.19	.019	1.049	.002	.986	-2.06 – 2.10
Baseline ADHD-RS total score[f]	.006	.047	.016	.897	-0.09 – 0.10	.006	.047	.016	.892	-0.09 – 0.10
Baseline WFIRS-P total score[f]	.065	.032	.242	.048*	0.001 - 0.13	.059	.032	.222	.067	-0.004 - 0.12
Response week 3 (≥ 20% sympt. reduction) n=56	-.921	.928	-.086	.323	-2.8 – 0.92					
Response week 12 (≥ 40% sympt. reduction) n=121						-1.495	1.171	-.109	.204	-3.81 – 0.82

[a]Model 1, R^2= 0,15, adj. R^2 = 0,08, [b]Model 2, R^2 = 0,15, adj. R^2 = 0,09

[c]≥ 1 comorbid diagnosis, [d]higher education, [e]any parental psychiatric diagnosis, [f]higher score, worse outcome

* p<0.05

Table 2c

	ADHD-RS hyp/imp score at 3-year follow-up									
	Model 1[a] Early treatment responders/responders at week-3					Model 2[b] Late treatment responders/responders at week-12				
Variable	**B**	**SE B**	**β**	**p**	**B 95%CI**	**B**	**SE B**	**β**	**p**	**B 95% CI**
Constant	2.245	2.66		.400	-3.01 – 7.50	3.469	2.854		.226	-2.18 – 9.12
Gender (female)	2.985	.972	.244	.003*	1.06 – 4.91	2.992	.978	.245	.003*	1.06 – 4.93
Age (10-12 yrs)	-2.41	.909	-.208	.009*	-4.21 – -0.61	-2.552	.913	-.221	.006*	-4.36 – -0.74
Comorbidity[c]	2.382	.905	.226	.010*	0.59 – 4.17	2.716	.888	.258	.003*	0.96 – 4.47
WISC-IQ (IQ > 85)	-.052	.974	-.004	.957	-1.98 – 1.88	-.070	.980	-.006	.943	-2.01 – 1.87
Maternal education[d]	-.279	.639	-.037	.663	-1.54 – 0.99	-.276	.643	-.036	.668	-1.55 – 0.10
Parental psychiatric disorder[e]	-1.012	.970	-.085	.299	-2.93 – 0.91	-1.162	.976	-.098	.236	-3.09 – 0.77
Baseline ADHD-RS total score[f]	.101	.043	.257	.021*	0.02 – 0.19	.104	.044	.265	.019*	0.02 – 0.19
Baseline WFIRS-P total score[f]	.009	.030	.034	.757	-0.05 - 0.07	-.001	.030	-.003	.978	-0.06 - 0.06
Response week 3 (≥ 20% sympt. reduction) n=56	-2.000	.856	-.185	.021*	-3.69 – -0.31					
Response week 12 (≥ 40% sympt. reduction) n=121						-2.172	1.089	-.157	.048*	-4.33 – -0.02

[a]Model 1, R^2= 0,28, adj. R^2 = 0,23, [b]Model 2, R^2= 0,27, adj. R^2 = 0,22

[c]≥ 1 comorbid diagnosis, [d]higher education, [e]any parental psychiatric diagnosis, [f]higher score, worse outcome

* $p<0.05$

Table 2d

	ADHD-RS behavior score at 3 year-follow-up									
	Model 1[a] Early treatment responders/responders at week-3					Model 2[b] Late treatment responders/responders at week-12				
Variable	**B**	**SE B**	**β**	**p**	**B 95%CI**	**B**	**SE B**	**β**	**p**	**B 95% CI**
Constant	-.630	2.463		.799	-5.50 – 4.25	.973	2.603		.709	-4.18 – 6.13
Gender (female)	1.051	.901	.092	.246	-0.73 – 2.84	1.021	.892	.089	.254	-0.74 – 2.79
Age (10-12 yrs)	-1.100	.843	-.101	.194	-2.77 – 0.57	-1.193	.833	-.110	.155	-2.841– 0.46
Comorbidity[c]	.978	.839	.099	.246	-0.68 – 2.64	1.087	.810	.110	.182	-0.52 – 2.30
WISC-IQ (IQ > 85)	-1.521	.903	-.137	.095	-3.31 – 0.27	-1.548	.894	-.139	.086	-3.32 – 0.22
Maternal education[d]	-.189	.593	-.026	.750	-1.36 – 0.98	-.153	.587	-.021	.795	-1.31 – 1.01
Parental psychiatric disorder[e]	.740	.899	.066	.412	-1.04 – 2.52	.627	.890	.056	.482	-1.13 – 2.39
Baseline ADHD-RS total score[f]	.086	.040	.233	.035*	0.01 – 0.17	.086	.040	.232	.034*	0.01 – 0.16
Baseline WFIRS-P total score[f]	.074	.028	.292	.009*	0.02 - 0.13	.067	.027	.265	.016*	0.01 - 0.12
Response week 3 (≥ 20% sympt. reduction) n=56	-1.109	.793	-.110	.165	-2.68 – 0.46					
Response week 12 (≥ 40% sympt. reduction) n=121						-2.136	.993	-.165	.034*	-4.10 – -0.17

[a]Model 1, R^2 = 0,30, adj. R^2 = 0,25, [b]Model 2, R^2 = 0,31, adj. R^2 = 0,26

[c]≥ 1 comorbid diagnosis, [d]higher education, [e]any parental psychiatric diagnosis, [f]higher score, worse outcome

* $p<0.05$

Table 2e

	WFIRS-P total score at 3 years follow-up									
	Model 1[a] Early treatment responders/responders at week-3					Model 2[b] Late treatment responders/responders at week-12				
Variable	**B**	**SE B**	**β**	**p**	**B 95%CI**	**B**	**SE B**	**β**	**p**	**B 95% CI**
Constant	10.246	10.570		.334	-10.69 – 31.17	10.621	11.322		.350	-11.79 – 33.04
Gender (female)	3.809	3.807	.083	.319	-3.73 – 11,35	3.991	3.838	.087	.301	-3.61 – 11.59
Age (10-12 yrs)	-5.702	3.596	-.130	.115	-12.82 – 1.42	-6.112	3.620	-.140	.094	-13.28 – 1.05
Comorbidity[c]	-.183	3.563	-.005	.959	-7.24 – 6.87	1.001	3.497	.025	.775	-5.92 – 7.93
WISC-IQ (IQ > 85)	-4.804	3.854	-.107	.215	-12.43 – 2.83	-4.842	3.889	-.108	.215	-12.54 – 2.86
Maternal education[d]	2.195	2.515	.077	.385	-2.78 – 7.17	2.129	2.539	.074	.403	-2.90 – 7.16
Parental psychiatric disorder[e]	1.931	3.791	.043	.611	-5.58 – 9.44	1.651	3.824	.037	.667	-5.92 – 9.22
Baseline ADHD-RS total score[f]	.037	.170	.025	.828	-0.30 – 0.37	.052	.171	.035	.760	-2.86 - 0.39
Baseline WFIRS-P total score[f]	.431	.117	.423	.000*	0.20 - 0.66	.404	.117	.401	.001*	0.176 – 0.64
Response week 3 (≥ 20% sympt. reduction) n=56	-5.499	3.358	-.136	.104	-10.84 – 2.66					
Response week 12 (≥ 40% sympt. reduction) n=121						-3.295	4-262	-.064	.441	-11.73 – 5.14

[a]Model 1, R^2= 0.23, adj. R^2 = 0.17, [b]Model 2, R^2= 0.22, adj. R^2 = 0.16

[c]≥ 1 comorbid diagnosis, [d]higher education, [e]any parental psychiatric diagnosis, [f]higher score, worse outcome

* $p<0.05$
